# Advances in HPV Vaccination in People Living with HIV: A Review

**DOI:** 10.3390/vaccines14020194

**Published:** 2026-02-21

**Authors:** Megan Mooberry, J. Brooks Jackson, Mary B. Rysavy

**Affiliations:** 1 Department of Pathology, Carver College of Medicine, University of Iowa, Iowa City, IA 52242, USA; megan-mooberry@uiowa.edu (M.M.); brooks-jackson@uiowa.edu (J.B.J.); 2 Department of Obstetrics, Gynecology, and Reproductive Sciences, McGovern Medical School, University of Texas Health Science Center at Houston, Houston, TX 77030, USA

**Keywords:** HPV vaccination, people living with HIV

## Abstract

Human papillomavirus (HPV) is the most common sexually transmitted infection worldwide and is a leading cause of cervical, anal, penile, and oropharyngeal cancers. This review summarizes the epidemiology of HPV and the immunogenicity, clinical efficacy, and current HPV vaccination recommendations among people living with HIV (PLWH). PLWH experience a disproportionate burden of HPV-related infection and HPV-related malignancies. Although HPV vaccines have been shown to be highly effective, vaccination coverage among PLWH remains suboptimal, particularly in low- and middle-income countries. Barriers to vaccination include extended dosing schedules, limited awareness of the vaccine, and misinformation. Evidence indicates HPV vaccines are safe and induce a robust antibody response in PLWH, especially among individuals with higher CD4+ cell counts and viral suppression on antiretroviral therapy. However, evidence for reduction in clinical HPV-related disease in this population remains limited. Ongoing research is aimed at optimizing the HPV vaccination schedule for PLWH and expanding vaccination in older, high-risk subgroups. Integrating HPV vaccination into HIV care is essential to reduce HPV-related morbidity and mortality in PLWH.

## 1. Introduction

Human papillomavirus (HPV) is a double-stranded DNA virus that infects basal epithelial cells in the skin and mucosal surfaces [1,2]. More than 200 HPV subtypes have been identified [3] and are broadly classified as low-risk or high-risk based on oncogenic potential. Low-risk strains, such as HPV 6 and 11, are not associated with malignancy, but are responsible for plantar warts, genital warts, and recurrent respiratory papillomatosis [4]. There are at least 14 high-risk strains, including HPV 16, 18, 31, and 45, which are associated with cervical, vaginal, vulvar, anal, penile, and oropharyngeal cancers [4].

HPV is transmitted primarily through skin-to-skin or mucosal contact and is the most common sexually transmitted infection worldwide [5]. The estimated individual lifetime risk of HPV exposure is between 50 and 80% [1]. The prevalence of HPV varies with age, screening practices, and geographic location, making population-level estimates challenging. A 2010 meta-analysis reported a global HPV prevalence of approximately 11% among women (6). Substantial geographic variation was observed with the highest prevalence rates reported in Eastern Africa at 24% and the lowest rates in North America at around 5% [6]. Across regions, HPV prevalence peaks in women under the age of 25 [6]. Estimating HPV prevalence in men remains challenging due to the absence of routine screening. However, a recent meta-analysis found a global HPV prevalence of 31% in men over the age of 15, including 21% infected with high-risk strains [7]. Known risk factors for HPV acquisition include multiple sexual partners, early sexual debut, prior sexually transmitted infection (STI), immunosuppression, and low socioeconomic status [1].

Most HPV infections are transient, with over 90% clearing spontaneously within 1–2 years of acquisition [8,9]. Risk factors for persistent infection include HPV subtype and viral load at the time of detection [8]. A small percentage of persistent infections progress to pre-cancerous or cancerous lesions.

HPV represents a substantial global health burden; it is estimated to account for 5% of all cancers worldwide [8]. HPV is implicated in over 90% of cervical and anal cancers. Additionally, it is responsible for an estimated 70% of vulvar and vaginal cancers, 60% of penile cancers, and 70% of oropharyngeal cancers [4]. High-risk HPV types 16 and 18 account for the majority of HPV-associated malignancies globally [10].

## 2. HPV and HIV Co-Infection

In 2023, an estimated 40 million people worldwide were living with human immunodeficiency virus (HIV) [11]. HIV prevalence remains disproportionately high in sub-Saharan Africa, particularly among young women aged 15–24, who accounted for approximately 25% of new HIV infections globally in 2017 [12,13]. Marked gender disparities persist, with HIV risk three to six times higher among young women compared with age-matched men across the region [13]. This region also experiences the highest incidence and mortality rates of cervical cancer across the globe [14,15].

**Figure 1:**
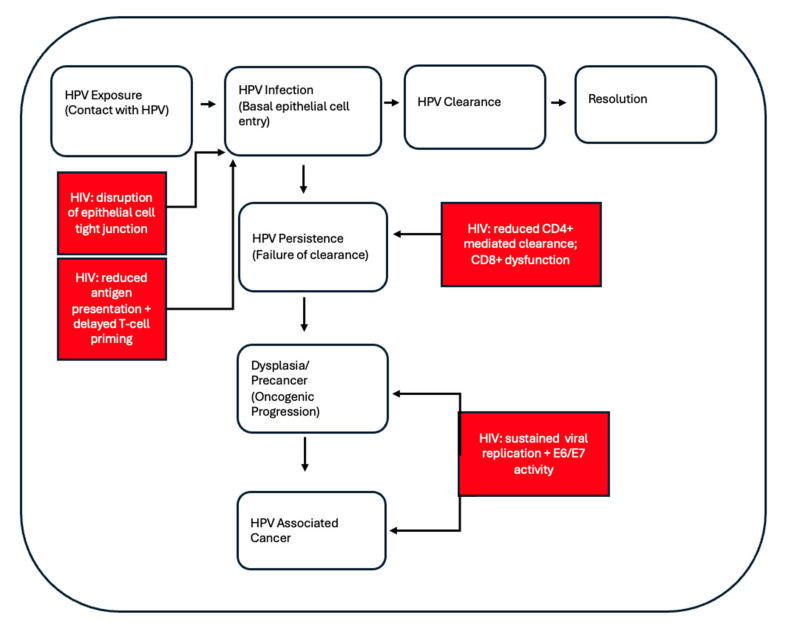
The impact of HIV on the Natural History of HPV Infection. Following exposure to HPV, most infections spontaneously resolve. In people living with HIV, disruption of the epithelial barriers, impaired antigen presentation, and delayed T-cell priming increase the risk of HPV persistence. Persistent infection promotes dysplasia and precancerous lesions. Sustained viral replication and reduced E6/E7 activity contribute to the progression of HPV-associated malignancy [24,25,26].

HPV infection is particularly prevalent among people living with HIV (PLWH) [16,17,18]. A large-scale metanalysis published in 2018 found that women with HIV had a significantly increased risk of acquiring HPV compared to HIV-negative controls (RR 2.64, 95% CI 2.04–3.42) [19]. When examining high-risk HPV genotypes, particularly HPV 16 and 18, prevalence rates among women living with HIV range from 40 to 60%, substantially exceeding rates observed in HIV-negative populations (23–38%) [19,20,21,22,23]. This increased susceptibility is driven in part by impaired cellular immunity, resulting in reduced viral clearance and increased HPV persistence [24,25]. At the cellular level, proteins secreted from HIV-infected intraepithelial immune cells cause disruption of epithelial cell tight junctions, which may facilitate HPV entry into target cells [24,26]. The impact of HIV infection on the natural history and progression of HPV infection is further summarized in Figure 1.

People living with HIV experience a higher burden of HPV-related malignancies than the general population [27]. Among women, HIV infection is associated with a 3 to 4-fold increased risk of cervical cancer [19,28,29,30]. This increased risk is likely driven by impaired clearance of high-risk HPV types and accelerated progression of precancerous lesions [28]. HIV-positive women also face elevated risks of other HPV-associated cancers, including oropharyngeal cancers (HR 2.55, 95% CI, 1.86–3.50) and anal cancers (HR 12.88 (95% CI, 8.69–19.07)) [31].

Among men, HIV similarly confers markedly increased risk across HPV-related malignancies. Studies show HIV infection in men is associated with a 20-fold higher risk of developing anal cancer [31]. HIV-positive men are also more likely to develop oropharyngeal cancers (HR 1.69, 95% CI, 1.39–2.04) and penile cancer (HR 0.63, 95% 1.64–4.23). [31,32]. Collectively, these elevated risks highlight the need for targeted screening, vaccination, and prevention strategies in HIV-affected populations.

## 3. HPV Vaccination

The first HPV vaccine was approved in 2006 across several countries, including Mexico, Australia, the United States, and parts of Europe [33]. As of 2025, 148 countries have incorporated HPV vaccination into their national immunization programs [34]. Three types of HPV vaccines are currently licensed and include bivalent, quadrivalent, and nonavalent forms [4]. The quadrivalent vaccine, Gardasil, was the first to be licensed and offers protection against HPV 6, 11, 16, and 18 [33]. Cervarix, a bivalent vaccine, offers protection against HPV 16 and 18 [33]. The only vaccine currently administered in the United States is the nonavalent vaccine, Gardasil 9, which provides protection against HPV 6, 11, 16, 18, 31, 33, 45, 52, 58 [35]. Globally, Gardasil 9 has largely replaced other vaccine types [36].

All vaccine types use virus-like particles (VLPs) that self-assemble into the L1 capsid protein of HPV [9]. This results in the production of a strong neutralizing antibody response without using viral DNA [37]. Importantly, HPV vaccines are prophylactic and do not clear existing infection, highlighting the importance of vaccination prior to HPV exposure.

The World Health Organization (WHO) recommends a routine one or two-dose vaccination regimen for girls aged 9–14 years, with catch-up vaccination available for girls through the age of 20 [38]. Universal vaccination for boys is not currently recommended by the WHO. In contrast, the Centers for Disease Control and Prevention (CDC) recommendations are broader. Prior to January 2026, the CDC advised a two-dose series for all girls and boys aged 11 to 12 (with the option to begin as early as age 9) [35]. In January 2026, in response to a previously issued Presidential Memorandum, the CDC updated its guidance to recommend a single dose vaccination series [39]. Catch-up vaccination is recommended through the age of 26 and may be offered up to age of 45 through shared clinical decision making [35]. Both organizations recommend a three dose vaccine series for individuals who are immunocompromised, including those living with HIV [35,40]. A comparison of the WHO and CDC HPV vaccination recommendations is summarized in Table 1.

The HPV vaccine consistently induces high seroconversion rates, with more than 99% of immunocompetent individuals developing detectable antibodies against vaccine-covered HPV types following vaccination [41,42]. The clinical efficacy of the HPV vaccine, typically defined as the prevention of persistent HPV infection, has been well studied, and is frequently reported between 90 and 100% when administered to HPV naïve women under the age of 26 [43,44,45,46,47]. This high level of protection is consistent across all licensed vaccine types [48]. In men, the quadrivalent vaccine has demonstrated an efficacy of 90% when administered to HPV naïve populations [44,49]. Studies have shown long-term protection following vaccination for at least 10 to 12 years [50,51].

The HPV vaccine has an excellent safety profile. Prior to licensure, randomized trials involving more than 50,000 women demonstrated no difference between vaccine and control groups in rates of serious adverse events, development of autoimmune or chronic disease, or death [52]. These findings have been confirmed by extensive post-licensure surveillance, with large population-based studies from multiple countries demonstrating no link between vaccination and adverse health outcomes [53,54,55,56]. The most commonly reported adverse events following vaccination are vasovagal syncope and injection-site reactions [55,57]. Injection-site reactions occur more frequently with nonavalent HPV vaccine compared with other vaccine formulations [58]. Anaphylaxis is rare, occurring at an estimated rate of 1.7 cases per million doses, and represents an absolute contraindication to further HPV vaccination [59].

Although the immunogenicity, clinical efficacy, and safety profiles of the HPV vaccine are well established in immunocompetent individuals, clinical efficacy data in PLWH are limited. Most studies of HPV vaccination in PLWH rely on seroconversion, the development of vaccine-induced antibodies, as a surrogate endpoint for clinical efficacy [27,60,61]. However, no definitive antibody threshold of protection against HPV has been established, making seroconversion an imperfect marker of clinical efficacy [61].

## 4. HPV Vaccination Coverage

In 2020, the WHO launched a global strategy to reduce cervical cancer, including a target of 90% HPV vaccination coverage among girls by the age of 15 by 2030 [62]. However, the most recent data (2023) suggests global coverage remains well below this benchmark. In countries with national vaccination programs, roughly 61% of women have received at least one dose of the HPV vaccine, and only about 48% of women have completed the full series [34]. Only eleven countries have reached the 90% full-dose coverage target. Coverage is slightly lower in men, with approximately 46% completing the recommended series [34]. In the United States, full-series coverage is reported at 64% in females and 59% in males [34,63].

Global disparities in HPV vaccination remain striking. Only 30% of low- and middle-income countries have national HPV vaccination programs, compared to over 80% of high-income countries [64]. Low- and middle-income countries experience over 90% of the global burden of cervical cancer deaths [65]. Lower vaccination coverage in these regions may be related to supply constraints, costs, and logistical challenges [64].

The Global Alliance for Vaccines and Immunization (GAVI) has supported pilot projects in 57 low-income countries to expand HPV vaccination coverage [66]. However, funding allocation is primarily based on national income classification, prioritizing the poorest countries [34]. As a result, this approach may lead to gaps in coverage in countries that do not meet eligibility thresholds but still experience substantial HPV burden.

Pilot programs supported by GAVI and other international organizations have been very effective, with studies demonstrating vaccination rates as high as 89% following program introduction [67,68]. However, following the withdrawal of initial pilot programs, significant declines in vaccination coverage have been observed, highlighting concerns regarding long-term sustainability [68].

Low- and middle-income countries that are not eligible for GAVI support or that have transitioned out of GAVI eligibility face unique challenges. Acquisition of the HPV vaccine accounts for an average of 22% of the total vaccine budget for non-GAVI eligible, middle-income countries [69]. This significant cost, combined with limited domestic funding in many settings, poses a major barrier to vaccination and may exacerbate vaccine inequities in regions with high HPV burden [69,70].

In an effort to increase vaccination coverage, there has been a shift towards a single-dose HPV vaccination strategy in many countries, even in the United States, as noted above [34]. Several studies have demonstrated that a single-dose regimen provides comparable protection to traditional two or three dose schedules [71,72,73]. The WHO updated its position in 2023 to include this recommendation, and as of 2025, 67 countries had adopted a single-dose schedule [34]. This approach has the potential to improve cost-effectiveness, simplify vaccine delivery, and expand vaccine coverage in resource-limited settings. However, it is important to emphasize evidence supporting the efficacy of a single-dose regimen in immunocompromised groups, including PLWH, remains limited. Efficacy of single dose HPV vaccine in PLWH may be more dependent on CD4+ cell count, degree of HIV viral suppression, frequency of HPV exposure, type of cART regimen, and/or type of HPV vaccine. Premature adoption of a single-dose schedule in these groups may risk suboptimal protection against HPV-associated disease.

## 5. HPV Vaccination Coverage in PLWH

There is limited research specifically evaluating HPV vaccination coverage among people living with HIV. Some studies suggest individuals living with HIV in the United States are more likely to receive at least one dose of the HPV vaccine as compared to the general population [74,75]. This trend may reflect more frequent engagement with healthcare services and targeted vaccination efforts within HIV care settings. In contrast, other studies show people living with HIV are less likely to be vaccinated compared to the general population, possibly due to older age at diagnosis and missed opportunities during routine adolescent vaccination programs [76,77].

Globally, HPV vaccination coverage among individuals living with HIV remains low. A recent study from Trinidad and Tobago involving more than 5000 people living with HIV found that only 21% had received at least one dose of the HPV vaccine [78]. Similarly, in Uganda, HPV vaccination coverage among female adolescents with HIV was limited, with only 31% receiving at least one dose and just 8% completing the full vaccination series [79]. A second study of adolescent girls receiving care at an HIV clinic in Kampala, Uganda, reported slightly higher uptake, with 48% of participants having received at least one dose of the HPV vaccine [80].

Lower vaccination rates in individuals living with HIV may be driven by several interconnected barriers. The three-dose HPV vaccination regimen recommended for people with HIV, compared to the one-two dose schedule recommended for the general population, may pose logistical challenges to series completion. A study of adolescents living with HIV in Uganda found many participants received their third dose of the vaccine at community outreach programs, whereas the first dose was most commonly administered via school-based programs [79]. This pattern suggests a gap in vaccine delivery programs, which are often designed for the general population and may not adequately accommodate the extended dosing schedules and follow-up needs specific to individuals living with HIV.

Knowledge deficits and misinformation also represent significant barriers to vaccination. In a study of men attending HIV clinics in Canada, 40% of participants were unaware of the HPV vaccine, and 65% perceived themselves to be at low risk for infection [81]. Similarly, a study of HPV vaccine uptake among HIV-positive adolescents in Uganda found that 40% were unaware of the vaccine, and 74% did not know the number of doses recommended for them to receive [79]. In Malawi, a study of female adolescents identified misconceptions linking the HPV vaccine to reduced fertility as a major deterrent to vaccination [82].

Strategies to improve HPV vaccination rates in people living with HIV, particularly in sub-Saharan Africa, include community-based approaches and targeted education. In Uganda, adolescent girls living with HIV who received counseling from a community member were three times more likely to receive the HPV vaccine series (OR 3.28, 95% CI 1.07–10.08). In contrast, counseling delivered by a healthcare worker was not associated with increased vaccine uptake compared with no counseling [79]. This finding suggests community-led interventions may be particularly effective in improving acceptance and access. Other studies report greater knowledge of HPV, the HPV vaccine, and the risks of cervical cancer as positively associated with higher vaccination rates [83,84]. For example, a study in northern Uganda found individuals who learned about cervical cancer at school had 13-fold higher odds of receiving the HPV vaccination, underscoring the importance of educational efforts [85]. For people living with HIV, who face a disproportionate burden of HPV-related disease, these strategies represent critical opportunities to improve prevention and reduce long-term cancer risk. Barriers to HPV vaccination in PLWH and potential strategies to improve vaccine uptake are summarized in Table 2.

## 6. Safety and Efficacy of HPV Vaccination in PLWH

The HPV vaccine has been shown to be safe and immunogenic in PLWH [77,86]. In terms of safety, HPV vaccination in this population is associated with low rates of adverse events, comparable to those observed in HIV-negative individuals. A meta-analysis of randomized controlled trials found no significant difference in overall adverse events (RR 1.0, 95% CI 0.9–1.2) or serious adverse events (RR 0.6, 95% CI 0.2–1.6) between vaccine and placebo groups [27]. The most commonly reported adverse event was injection site reaction, occurring in approximately 20–25% of all cases, and was typically mild to moderate in severity [86,87,88]. This is similar to the rate of injection site reaction reported following vaccination in immunocompetent patients (25–40%) [89].

Immunogenicity data are similarly reassuring. The quadrivalent vaccine demonstrates seroconversion rates ranging from 91% to 96% following vaccination, which is comparable to rates observed in HIV-negative populations [27,61,88,90]. Although antibody titers tend to be lower in HIV-positive individuals compared with age-matched controls, the clinical significance of this difference remains unclear [61,88]. Notably, durable antibody responses have been observed, with some studies demonstrating persistent antibody titers for at least five years in HIV-positive adolescents following vaccination [91,92].

Vaccine immunogenicity in people living with HIV appears to be closely linked to immune status, particularly CD4+ cell count. In the AIDS Clinical Trials Group study of 319 HIV-positive women who received the quadrivalent HPV vaccine, seroconversion rates were highest among individuals with CD4+ counts > 350 cells/µL, reaching 91–99% across vaccine-covered HPV types [93]. In contrast, individuals with CD4+ counts < 200 cells/µL demonstrated lower serologic responses, with seroconversion rates ranging from 75 to 93% [93]. These findings suggest that higher CD4+ levels are associated with stronger vaccine responses in HIV-positive populations, mirroring clinical observations where lower CD4+ counts are associated with persistent infection and HPV-related disease [94].

Consistent with this relationship, several studies have reported higher seroconversion rates in individuals receiving combination anti-retroviral therapy (cART) compared to those not on therapy [95,96]. In a study of 99 women with HIV, seroconversion rates reached 100% for all quadrivalent HPV types among those receiving cART, while rates in the non-cART group ranged from 92.3% (HPV 18) to 100% (HPV 6). These findings emphasize the importance of viral suppression in optimizing vaccine response [96].

Despite robust safety and immunogenicity data, evidence related to clinical efficacy remains more limited. In one cohort of vaccinated girls and women living with HIV, the incidence rate of persistent quadrivalent HPV infection was 2.3 per 100 person-years, higher than the rate observed in vaccinated women without HIV (0.1 per 100 person-years) [94]. Among men, findings have been mixed. A randomized controlled trial of men living with HIV over the age of 27 found no significant protection against new anal HPV infections [97]. In contrast, a study in younger HIV-positive men who were HPV-naïve at baseline demonstrated protection against vaccine-type infection [98]. These findings suggest age and prior HPV exposure play a significant role in vaccine effectiveness. Overall, additional population-level studies are needed to better define clinical vaccine efficacy in HIV-positive populations.

Following vaccination, antibody titers in individuals with HIV gradually decline, similar to the trends observed in HIV-negative populations [61]. Some studies suggest that administering a fourth dose of the HPV vaccine, rather than the standard three dose regimen, may enhance long-term immunity, though this approach has yet to be widely adopted [99]. Evidence of the effectiveness of a two-dose regimen in people with HIV remains limited and inconclusive [61]. The safety and immunogenicity of the HPV vaccine in PLWH as compared to HIV-negative individuals is summarized in Table 3.

## 7. Update on Current Research

Ongoing research into HPV vaccination in people living with HIV reflects a growing focus on optimizing vaccination strategies for this population. Traditional three-dose schedules have demonstrated acceptable immunogenicity and safety profiles. However, emerging evidence has highlighted the effect of CD4+ count and cART suppression on vaccine response. Consequently, reduced-dose vaccination strategies are an active area of investigation. Clinical trials, such as HOPE-II, are evaluating whether a single dose of the nonavalent HPV vaccine can elicit a sustained immune response in women living with HIV [100]. Another ongoing study, NOVA-HIV is assessing the immunogenicity and safety of a reduced schedule of the nonavalent vaccine, which may be particularly useful in low-resource settings or for those with limited access to healthcare [101].

It is important to note, these strategies have yet to be validated on a widespread scale. Future research is necessary to identify which subgroups may benefit from receiving a reduced vaccine schedule. Factors such as age, antiretroviral therapy use, and CD4+ cell count may influence the decision to administer fewer vaccine doses.

Another area of active research involves extending HPV vaccination recommendations to older age groups. A recent trial conducted in Spain evaluated the nonavalent vaccine in HIV-positive men who have sex with men up to the age of 35. Results were encouraging, with over 85% of participants achieving seroconversion by week 96. Older age (>26) and lower CD4+ count were not associated with lower immunogenicity, suggesting potential benefit of vaccination in older, high-risk populations [102]. Of note, participants in the study had been on cART for a median of 4 years and had a median CD4+ count of 726 cells/µL, which may limit the generalizability of study findings [102].

Despite these advancements, substantial research gaps remain. Clinical efficacy data for HPV vaccination in HIV-positive populations are limited, with most studies focusing on immunogenicity rather than the prevention of persistent infection or HPV-related disease. The lack of large randomized controlled studies with clinical endpoints limits definitive conclusions regarding vaccine effectiveness in this population, despite consistent evidence of robust immune response.

## 8. Conclusions

HPV remains a major global health burden, accounting for approximately 5% of cancers worldwide [7]. People living with HIV experience significantly higher rates of HPV acquisition, persistence, and progression to HPV-related malignancies. 

Although HPV vaccination programs have expanded globally, coverage remains inconsistent, and vaccine uptake in individuals with HIV varies widely across settings, with particularly low coverage in low- and middle-income countries. Existing evidence demonstrates that HPV vaccines are safe and immunogenic in PLWH, with comparable seroconversion rates to those observed in HIV-negative populations, especially among those with higher CD4+ cell counts and viral suppression on cART. However, data on clinical effectiveness, particularly on the prevention of persistent infection and HPV-related disease, remain limited.

Ongoing research aims to optimize HPV vaccination strategies for individuals living with HIV, including the potential role of reduced-dose regimens and the extension of vaccination to older or previously exposed individuals. Continued investigation is essential to improve vaccination coverage and reduce the disproportionate burden of HPV-related disease among people living with HIV.

## Figures and Tables

**Table 1:** Comparison of WHO and CDC HPV vaccination recommendations. Both organizations emphasize early vaccination prior to HPV exposure and expanded dosing for immunocompromised individuals, including people with HIV.

Category	WHO Guidelines	CDC Guidelines
Target Age	Girls aged 9–14 years	All adolescents aged 11–12 years (can start at age 9)
Catch-Up Vaccination	Girls up to the age of 20	Up to 26 years of age
Adults > 26 years old	Not routinely recommended	Shared clinical decision making for age 27–45 years
Dose Schedule (Immunocompetent)	1–2 doses	1–2 doses
Dose Schedule (Immunocompromised)	3 doses recommended	3 doses recommended
Vaccine Type	Bivalent, quadrivalent, or nonavalent depending on availability	Nonavalent

**Table 2:** Barriers to and Strategies for Improving HPV Vaccination Coverage among People Living with HIV (PLWH). Barriers to HPV vaccination occur at both the individual and systemic level. Corresponding strategies emphasize education, community outreach, and integration of care to improve uptake of the HPV vaccine.

**Patient Level Barriers** **Strategies for Improvement**	
Limited awareness of the HPV vaccine	Targeted HPV education on the HPV and the vaccine for PLWH
Misperception of personal risk for HPV infection	Risk-focused counseling and education
Lack of knowledge about dosing requirements for PLWH	Clear communication of vaccine schedule
Difficulty completing the 3-dose schedule	Mobile clinics and outreach programs
**System Level Barriers** **Strategies for Improvement**	
Vaccine cost and supply limitations	Subsidized vaccine programs
Fragmented care delivery and limited follow up for extended vaccine schedules	Integration with HIV treatment services and community outreach programs

**Table 3:** Safety and Immunogenicity of HPV vaccines in People living with HIV (PLWH). The HPV vaccine is highly immunogenic and demonstrates a favorable safety profile in PLWH. However, data regarding the clinical efficacy of the vaccine in PLWH is limited. * Seroconversion rates following vaccination in PLWH are closely linked to CD4+ cell count and viral suppression on cART.

	PLWH	HIV-Negative Individuals
Seroconversion Rates	91–95% *	>99%
Antibody Titers following Vaccination	Lower	Higher
Durability of Antibody Titers	At least five years	At least 10–12 years
Clinical Efficacy	Not well studied	90–100%
Adverse Effects	Few; injection site reaction is the most common	Few; injection site reaction is the most common

## Data Availability

No new data were created or analyzed in this study.

## Abbreviations

The following abbreviations are used in this manuscript:

HPV	Human Papillomavirus
HIV	Human immunodeficiency virus
PLWH	People living with HIV
VLP	Virus-like particle
WHO	World Health Organization
CDC	Center for Disease Control and Prevention
GAVI	Global Alliance for Vaccines and Immunization
cART	Combined anti-retroviral therapy
